# An Unusual Presentation of Pubic-type Anterior Hip Dislocation with Concomitant Anterior and Posterior Acetabular Wall Fracture

**DOI:** 10.7759/cureus.4390

**Published:** 2019-04-05

**Authors:** Mayur Nayak, Rahul Yadav, Binay K Sahu, Venkatesan S Kumar, Vijay Sharma

**Affiliations:** 1 Orthopaedics, All India Institute of Medical Sciences, New Delhi, IND

**Keywords:** anterior hip dislocation, acetabular wall fracture, pubic type, open reduction

## Abstract

Anterior hip dislocation is uncommon, comprising only 5%-15% of hip dislocations. It usually occurs following a severe external rotation and abduction injury. These injuries are occasionally associated with acetabular fractures, which generally occur in the direction of dislocation. We describe a rare case of pubic-type anterior hip dislocation with concomitant anterior and posterior acetabular wall fractures in a young male following a road traffic accident. The dislocation could not be reduced by closed means and open reduction had to be performed. Reduction of the hip allowed the wall fragments to fall back to their place and the hip remained stable. At the one-year follow-up, the clinical and radiological results were excellent. This case also emphasizes the importance of early diagnosis and prompt reduction in the successful management of these types of injury.

## Introduction

Traumatic hip dislocation is an extremely severe injury caused due to high-energy trauma such as a road traffic accident. It has increased in recent years due to the increase in the number of motor vehicle accidents [1]. These injuries are relatively uncommon, constituting about 5% of all dislocations [1]. Hip dislocations can be classified into anterior, posterior, and central, depending upon the position of the head in relation to the acetabulum. Anterior hip dislocations account for about 10% of hip dislocations [2-4]. Such dislocations are occasionally associated with acetabular fractures [3,5-7]. Early diagnosis and prompt reduction form the cornerstone for the treatment.

## Case presentation

**Figure 1:**
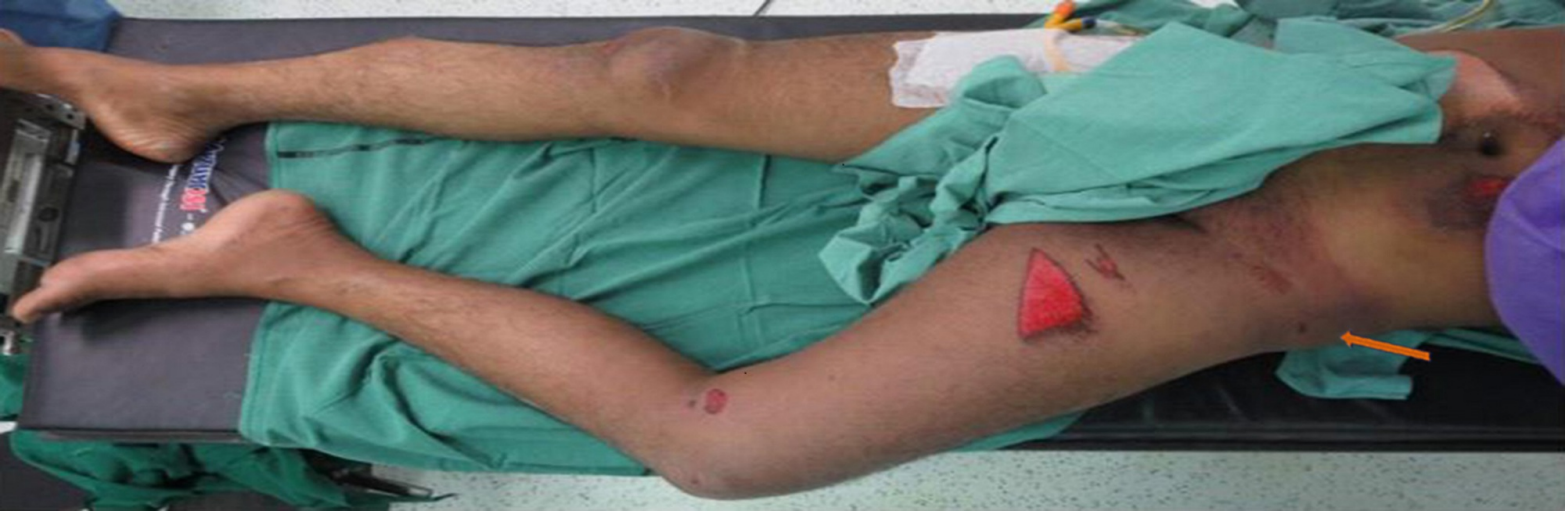
Characteristic deformity Characteristic deformity of the limb with shortening and external rotation of the left hip in the emergency department

**Figure 2:**
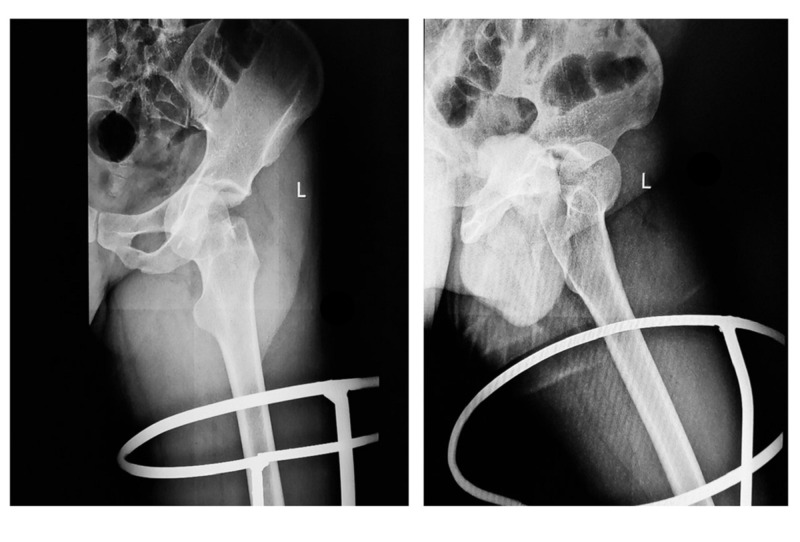
AP and lateral X-ray Anteroposterior (AP) and lateral X-ray of left hip showing pubic-type anterior dislocation of the left hip

**Figure 3:**
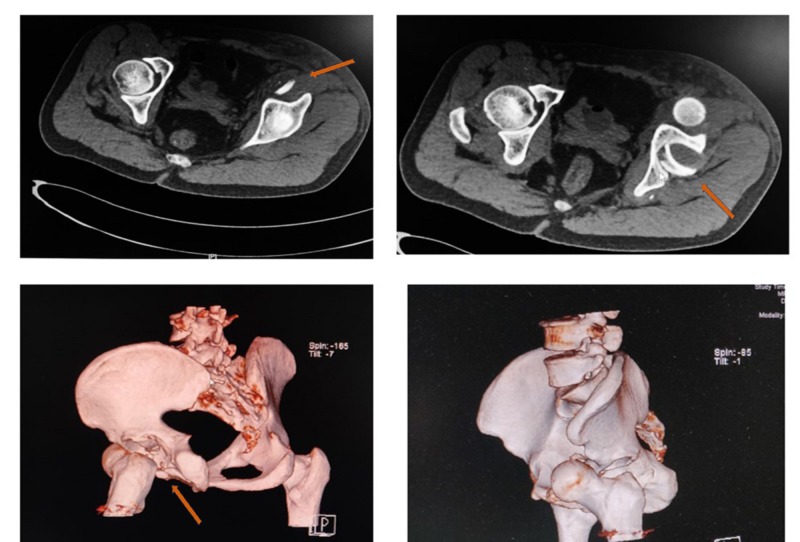
Preop CT imaging Pre-reduction coronal and 3D computed tomography (CT) scan of the left hip with the pelvis showing anterior dislocation of the left hip with the fracture of the anterior wall and the posterior wall of the acetabulum

**Figure 4:**
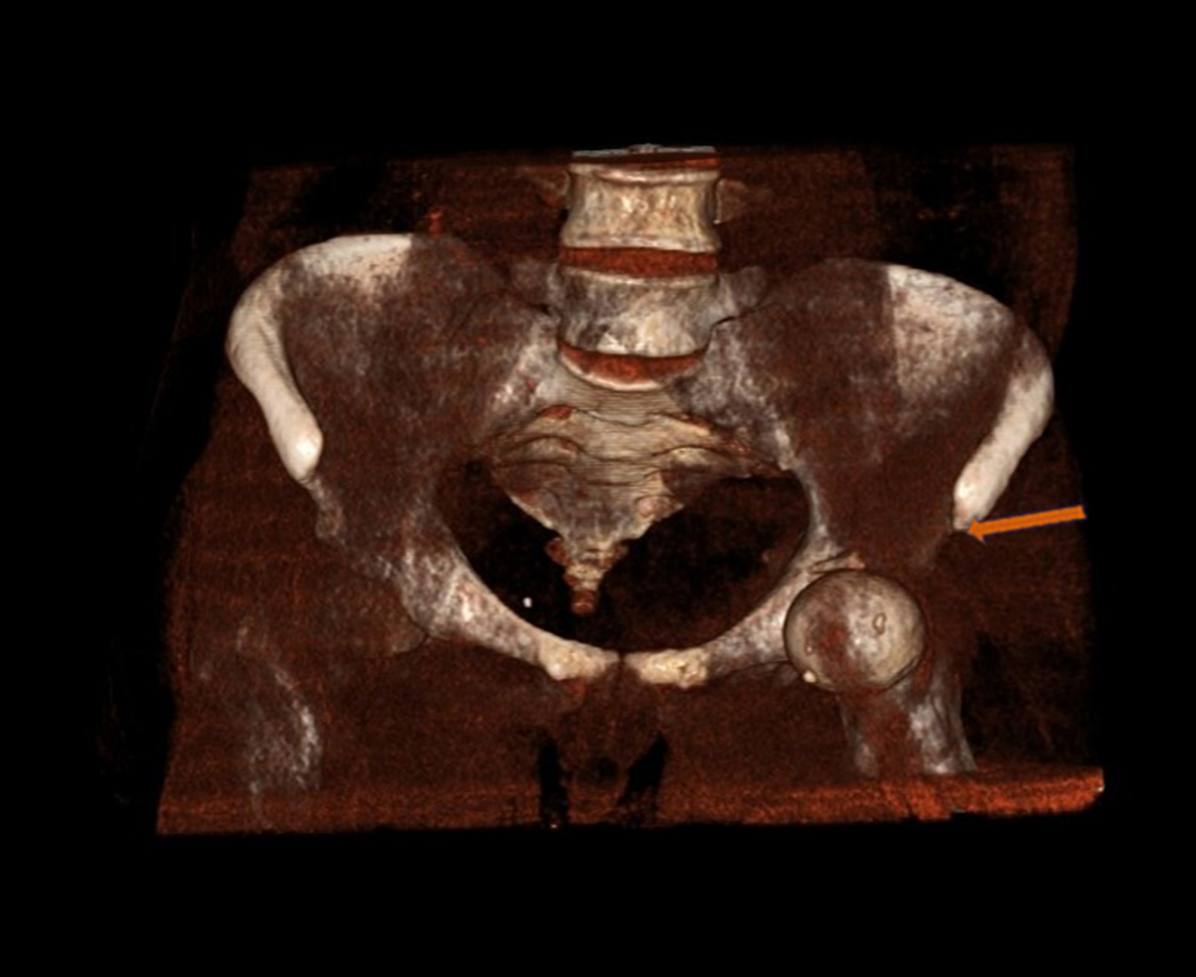
Trapped femoral head The left femoral head trapped in the iliopsoas

**Figure 5:**
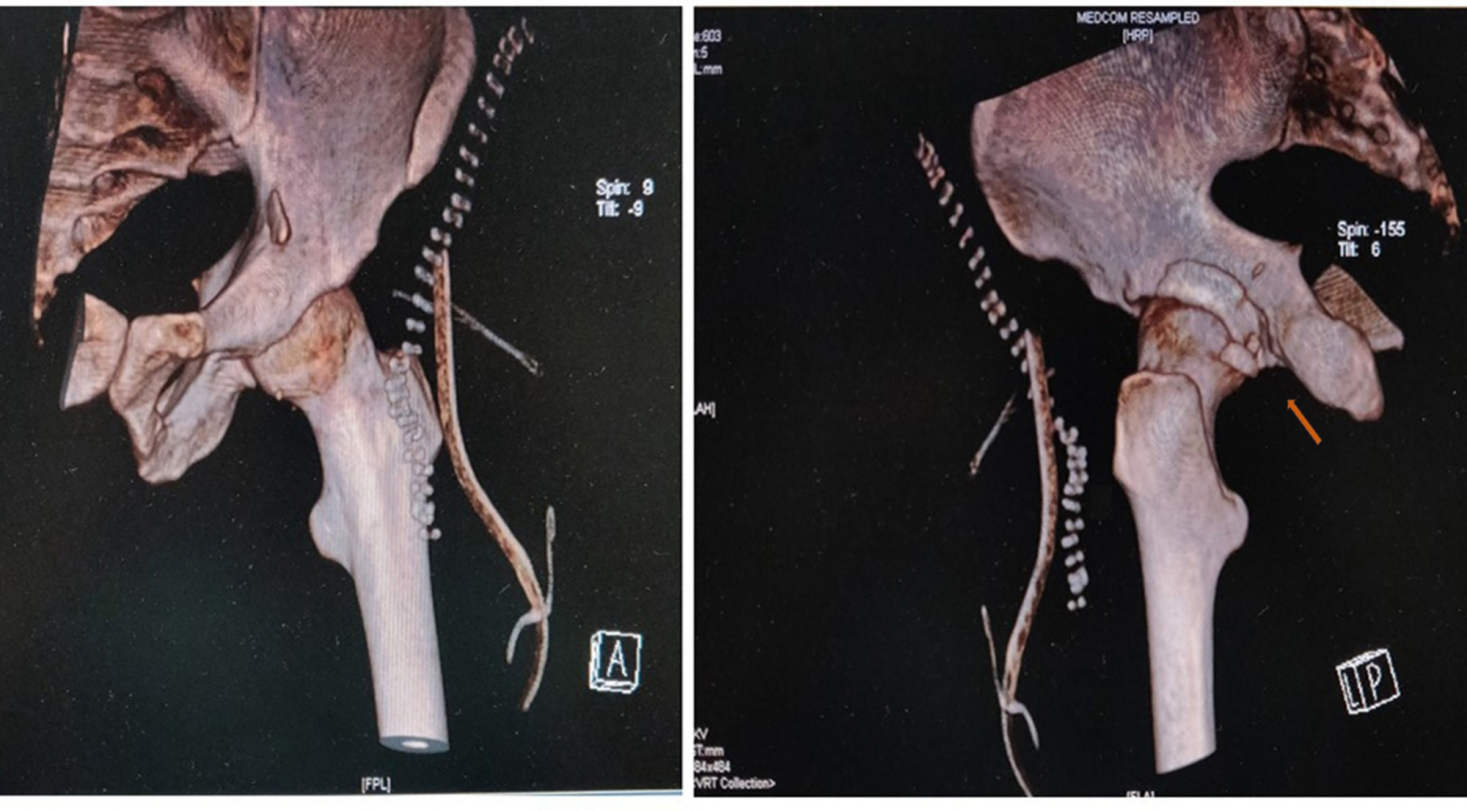
Post-reduction computed tomography (CT) Three-dimensional (3D) cut section of the left hip with pelvis showing the complete reduction of the posterior wall fragment following open reduction

**Figure 6:**
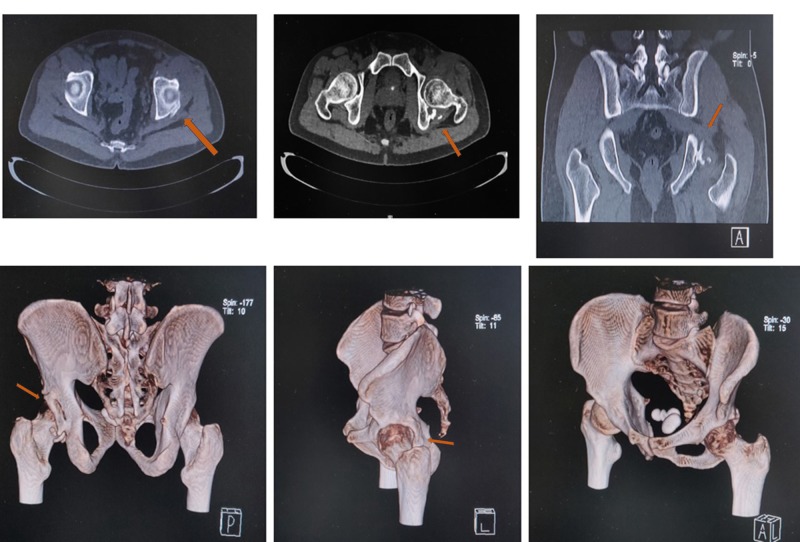
CT imaging at one year Coronal, axial, and three-dimensional (3D) section of the computed tomography (CT) scan at one year, showing the complete bony union of the anterior and posterior walls of the acetabulum

A 21-year-old male presented to our emergency department (ED) following a high-velocity road traffic accident (RTA), sustaining an injury to his left hip. While riding his motorcycle, he lost control of his bike and fell on his left hip. Immediately, he felt severe pain in the left hip and was unable to bear weight over the left hip. On arrival to the ED, the patient was managed according to the advanced trauma life support (ATLS) protocol. The left lower limb was grossly shortened and externally rotated (Figure 1). Swelling was noted in the left hip and thigh region. Tenderness was present near the hip joint and movements were painful. An abrasion of approximately 10 cm x 15 cm was present over the medial aspect of the left thigh. The neurovascular status was normal. Standard radiographs showed an anterior dislocation of the hip with an intra-articular bone fragment (Figure 2). After stabilizing the vitals, the patient underwent a computed tomography (CT) scan of the pelvis, with both hips, which showed an anteriorly dislocated femoral head lying externally rotated in relation to the superolateral aspect of the acetabulum. In addition, a displaced posterior wall fracture fragment was found within the acetabulum and a separate anterior wall fracture was noted (Figure 3). The patient was immediately rushed to the operation theater after consenting for both closed and open reduction. After general anesthesia, closed reduction was attempted by the Allis method under image guidance. The femoral head could not be displaced from its position despite three attempts and a decision was made to proceed with open reduction using the Smith-Peterson approach. It was found that the head was trapped in the iliopsoas muscle and the detached fragment was found to be the posterior acetabular wall (Figure 4). The capsule was found to be torn. Open reduction was performed and the position of the posterior wall was restored (Figure 5). The anterior wall, being a very small fragment, did not need reduction. The walls' fragments were not fixed, as they were completely reduced and the hip remained stable after reduction. Rehabilitation consisted of on-traction mobilization exercises of the hip in bed. At six weeks, non-weight bearing mobilization was initiated with the help of crutches. By 12 weeks, the fracture showed signs of union and partial weight-bearing mobilization was started. At the end of one year, the patient was independently mobile and was able to sit cross-legged, squat, and use public transport. The outcome was assessed by using the Modified Harris Hip score at six months and one year, which was 66 points at six months and 87 points at one year. The CT images at the one-year follow-up showed complete union (Figure 6).

## Discussion

The majority of hip dislocations are posterior dislocations following a dashboard injury [3,8-9]. Anterior hip dislocations are rare and usually occur following a severe external rotation and abduction injury. Sahin et al. reviewed 62 cases of hip dislocation in their study and found only five cases of anterior dislocation [1]. The dislocation of the head of the femur occurs in the direction of the applied force. Epstein classified anterior hip dislocations based on the position of the femoral head: superior and inferior. Superior dislocation can be classified into pubic and iliac, which can be further subdivided into three types based on the associated fractures, Type A (no fracture), Type B (associated femoral head fracture), and Type C (associated acetabular fracture) [10-11]. The dislocation of the hip depends upon the direction of the force and the position of the hip. Flexion of the hip will result in either a perineal or an obturator dislocation while an extension of the hip will result in a pubic type of dislocation. Inferior hip dislocations are more common. The head typically displaces through the pubofemoral and ischiofemoral ligaments and comes to rest upon the obturator ring. There can be associated anterior wall fractures, however, they occur infrequently [8].

Associated injuries, such as femoral head fractures and osteochondral defects, are common, whereas other injuries, such as acetabular fracture, femoral neck fractures, greater trochanter, and femoral shaft, are rare [5]. Alonso et al. [8] conducted a retrospective analysis of 88 hip dislocations associated with acetabular fractures and found only 2% of cases with anterior dislocation. Epstein et al. [9], in their series of 55 patients with anterior hip dislocation, found only two cases of associated acetabular fractures. Buckwalter et al. [12] presented a historical review of 104 cases of asymmetrical bilateral hip dislocations and found that there were 53 associated acetabular fractures, 39 associated with posterior dislocations, and 12 with anterior dislocations. In all the above-mentioned series, the details of acetabular fractures were not described.

As a norm, acetabular wall fractures associated with hip dislocations occur in the direction of dislocation, i.e., anterior wall fractures are associated with anterior hip dislocations while posterior wall injuries are common with posterior hip dislocations. There have been few reports in the past describing the anterior dislocation of the femoral head in association with anterior wall fractures [2,5-6]. Chadha et al. [13], on the other hand, reported an uncommon presentation of the iliac type of anterior hip dislocation associated with a posterior acetabular wall fracture. Previously, a case of acetabular fracture with a similar fracture pattern was described by Chen et al. [14]; however, it was associated with a posterior dislocation of the hip. In this report, we present a unique case of the pubic type of anterior dislocation of the hip with a fracture of the anterior wall as well as the posterior wall. In our opinion, this could have resulted from a possible dynamic injury leading to axial loading of the femur in flexion, thereby causing a posterior acetabular wall fracture. However, before posterior dislocation of the hip could have occurred, a probable change in the direction of the acting force pushed the hip into extension, external rotation, and abduction, causing an additional anterior wall fracture along with subsequent anterior dislocation of the head.

Anterior hip dislocation usually presents as a part of polytrauma and may be easily missed [8]. So, it is of utmost importance to perform a detailed clinical evaluation and proceed according to the ATLS protocol in these patients. An anteroposterior radiograph is an absolute necessity when a pelvic injury is suspected. It is important to see the lesser trochanter morphology, which acts as a guide to diagnosis [5]. Prompt reduction of the dislocated hip is necessary to obtain favorable results. When dislocations are not addressed within six hours of the injury, a poor outcome has been reported [15]. Reduction is generally achieved by closed means. Anterior dislocation of the hip is best reduced under general anesthesia with the patient in the supine position. Various maneuvers have been described for the same [10,16-19]. A CT scan must be obtained immediately after reduction to look for associated injuries, such as intra-articular loose bodies, acetabular fractures, femoral head fractures, and congruent reduction [5]. Open reduction is required if there is a hindrance to the reduction of the femoral head, such as entrapment of the head in the iliofemoral and pubofemoral ligaments [6], trapping of the head by the iliopsoas [7], or buttonholing of the capsule by the head [20]. In the present case, we found that the iliopsoas was passing over the femoral neck and attempted traction made the tendon taut, preventing reduction of the femoral head. Identifying this pattern is important, as a closed reduction is not feasible and an early open reduction is recommended in such injuries.

Early diagnosis and stable reduction form the cornerstone of successful management in these injuries. Late recognition and management lead to complications, the most common being osteoarthritis and avascular necrosis [5,15].

## Conclusions

This case highlights an unusual combination of an acetabular fracture associated with anterior hip dislocation, tries to explain the mechanism of the injury, and emphasizes the importance of early recognition and prompt reduction. The understanding of the buttonhole mechanism of the iliopsoas in the pubic type dislocation is important, as the closed reduction method is less feasible and a posterior wall fracture with an anterior hip dislocation may not require surgical fixation if the hip joint is stable.
